# Surveying the management of Achilles tendon ruptures in the Netherlands: lack of consensus and need for treatment guidelines

**DOI:** 10.1007/s00167-018-5049-5

**Published:** 2018-07-03

**Authors:** Olivier C. Dams, Inge van den Akker-Scheek, Ron L. Diercks, Klaus W. Wendt, Johannes Zwerver, Inge H. F. Reininga

**Affiliations:** 1 Department of Sport and Exercise Medicine, University of Groningen, University Medical Center Groningen, Hanzeplein 1, 9713 GZ Groningen, The Netherlands; 2 Department of Orthopaedics, University of Groningen, University Medical Center Groningen, Hanzeplein 1, 9713 GZ Groningen, The Netherlands; 3 Department of Trauma Surgery, University of Groningen, University Medical Center Groningen, Hanzeplein 1, 9713 GZ Groningen, The Netherlands

**Keywords:** Achilles, Rupture, Epidemiology, Ankle injuries, Treatment, Diagnostics, Clinical protocol, Traumatology, Orthopaedics, Surgical procedures, Rehabilitation

## Abstract

**Purpose:**

This study aimed to describe and analyse usual care of Achilles tendon ruptures (ATRs) by orthopaedic surgeons and trauma surgeons in the Netherlands.

**Methods:**

A nationwide online survey of ATR management was sent to all consultant orthopaedic and trauma surgeons in the Netherlands, requesting participation of those involved in ATR management. Data on individual characteristics and the entire ATR management (from diagnosis to rehabilitation) were gathered. Consensus was defined as ≥ 70% agreement on an answer.

**Results:**

A total of 91 responses (70 orthopaedic surgeons and 21 trauma surgeons) were analysed. There was consensus on the importance of the physical examination in terms of diagnosis (> 90%) and a lack of consensus on diagnostic imaging (ultrasound/MRI). There was consensus that non-surgical treatment is preferred for sedentary and systemically diseased patients and surgery for patients who are younger and athletic and present with larger tendon gap sizes. There was consensus on most of the non-surgical methods used: initial immobilisation in plaster cast with the foot in equinus position (90%) and its gradual regression (82%) every 2 weeks (85%). Only length of immobilisation lacked consensus. Surgery was generally preferred, but there was a lack of consensus on the entire followed protocol. Orthopaedic and trauma surgeons differed significantly on their surgical (*p* = 0.001) and suturing techniques (*p* = 0.002) and methods of postoperative immobilisation (*p* < 0.001). Orthopaedic surgeons employed open repair and Bunnell sutures more often, whereas trauma surgeons used minimally invasive approaches and bone anchors. Rehabilitation methods and advised time until weight-bearing and return to sport varied. Orthopaedic surgeons advised a significantly longer time until return to sport after both non-surgical treatment (*p* = 0.001) and surgery (*p* = 0.002) than trauma surgeons.

**Conclusion:**

This is the first study to describe the entire ATR management. The results show a lack of consensus and wide variation in management of ATRs in the Netherlands. This study shows that especially the methods of the perioperative and rehabilitation phases were inconclusive and differed between orthopaedic and trauma surgeons. Further research into optimal ATR management regimens is recommended. In addition, to achieve uniformity in management more multidisciplinary collaboration between Dutch and international surgeons treating ATRs is needed.

**Level of evidence:**

Cross-sectional survey, Level V.

**Electronic supplementary material:**

The online version of this article (10.1007/s00167-018-5049-5) contains supplementary material, which is available to authorized users.

## Introduction

Although the Achilles tendon is capable of tolerating heavy loading, it can be susceptible to injury, as the most frequently ruptured tendon [1]. The incidence of Achilles tendon rupture (ATR) has steadily increased in the Western world over the past years [2–5] and is expected to rise further, especially among the middle-aged, likely due to higher rates of recreational sports participation and obesity [6–9]. In addition to the rising incidence, ATRs can significantly burden patients, with more than half showing functional deficits and/or reporting pain even 12 months after injury, and many unable to return to their pre-injury level of activity [2, 10–12].

Despite these figures, a clear international management consensus for the treatment of ATRs is lacking. The only available guidelines are those of the American Academy of Orthopedic Surgeons (AAOS) and have limited or inconclusive recommendations for the role of imaging, choice of treatment (non-surgical/ surgical) and rehabilitation methods as a result of either lacking or unconvincing scientific evidence [13]. In addition, neither the UK’s National Institute for Health and Care Excellence (NICE), the British Orthopaedic Association, the Nordic Orthopaedic Federation, the Dutch Society for Orthopaedic Surgery (NOV) nor the Dutch Society for Trauma Surgery (NVT) has published evidence-based guidelines for the management of ATRs. Because of this lack of evidence, we hypothesise that all phases of ATR management in the Netherlands lack consensus; this leads to a divergent clinical protocol and potentially variation in outcome for patients.

Two studies have described multiple phases of reported ATR management in the United Kingdom (UK) and Scandinavia, concluding that there is a wide variation in treatment options that practitioners adhere to and that clear consensus is lacking [14, 15]. However, no such studies have been conducted in the Netherlands where ATRs are treated by both trauma and orthopaedic surgeons.

The aim of this study was to describe and analyse usual ATR care in the Netherlands, to create transparency about how this injury is being managed without clinical guidelines. Differences within and between the two treating specialisms were also compared and contrasted. This is the first study to describe the entire state of practice and the first to do so in the Netherlands. The analysis will highlight the barriers to achieving clinical guidelines, guide future research directions and allow clinicians to reflect on and compare their ATR treatment.

## Materials and methods

### Study design

Design of this study was a cross-sectional survey of practice. The local ethics committee of the University Medical Center Groningen judged the methods employed and waived further need for approval (METc #2016.475).

### Study population

All consultant orthopaedic surgeons (*n* = 601) and trauma surgeons (*n* = 230) registered in their respective Dutch specialist societies [the Dutch Societies for Orthopaedic Surgery (NOV) and Trauma Surgery (NVT)] were approached as potential study participants. Within these groups, those surgeons actually treating ATRs in their respective hospitals were asked to participate by filling in an online survey about ATR management. We estimate that the study population of surgeons actually treating ATRs consists of approximately 150 orthopaedic surgeons and 50 trauma surgeons, based on one or two surgeons per hospital treating ATRs.

### The survey

The survey was developed by epidemiologists, orthopaedic surgeons, trauma surgeons and sport and exercise medicine physicians with relevant clinical and methodological expertise (OCD, IvdAS, RLD, KWW, JZ, IHFR). It was designed to assess responders’ characteristics and the complete applied/preferred management decisions from diagnosis to rehabilitation. In designing the questions, attention was paid to recommendations made in the AAOS guidelines [13]. The survey consisted of a minimum of 21 and a maximum of 35 items, depending on the type of ATR treatment (non-surgical, surgical or both) responders use in their practice. Most questions were presented in multiple-choice format. The Appendix describes the survey questions.

Items in the survey were divided into four major sections: (1) individual characteristics, (2) diagnostic tools used, (3) preferred/applied primary treatment of ATRs, and (4) rehabilitation methods and advised time until return to sport (RTS). In each section responders were asked questions about:

1. Their medical specialism, years of experience, practice setting and number of ATRs treated per year.
2. Use of diagnostic tools [physical examination: palpation of tendon gap, Thompson test [16]; imaging: ultrasound (US), MRI, X-ray and CT] for which responders were asked to select all they apply for diagnosis and treatment-planning.
3. Type of preferred primary treatment (non-surgical or surgical) in the context of the individual patient, dependent on clinical factors such as age, American Society of Anesthesiologists (ASA) physical status, body mass index (BMI), activity level, tendon gap size and time between presentation and injury. Responders had to report whether they had a general preference for non-surgical or surgical treatment or whether they treated one way or the other exclusively. Subsequently, they were presented with an online pathway asking about the non-surgical and/or surgical treatment methods used.
4. Methods of rehabilitation, referral to physiotherapists, time advised for commencement of return to sport (RTS) and a selection of multiple outcomes (patient follow-up, questionnaires, physical tests, imaging) deemed relevant when monitoring recovery. For this study, the start of the rehabilitation phase was defined as the period after initial immobilisation in non-surgical patients and 6 weeks after surgery in surgical patients. Rehabilitation methods and advised time to RTS were asked about after both non-surgical and surgical treatment.

### Survey administration

Consultant surgeons were approached through the regular electronic newsletters of their Dutch specialist associations, NOV and NVT. The newsletters contained a brief description and a hyperlink to the survey. Although the survey was sent to all consultant surgeon members, it asked exclusively for participation of surgeons actually treating ATRs. The survey was sent out once to the NOV in December 2016 and twice to the NVT in December 2016 and January 2017.

In the online survey, environment responders were presented with a distinct pathway concerning only the respective treatment methods (non-surgical and/or surgical) they use. This pathway was generated by the question about the general preferred treatment, for which responders could indicate if they only treated surgically or only treated non-surgically. Data of the survey were stored on the server of the University Medical Center Groningen.

### Statistical analysis

Data were recorded on a tabulated form on Microsoft Excel 2010. All the required variables were converted to IBM SPSS Statistics for Windows software (Version 23.0, Armonk, NY: IBM Corp.) for statistical analysis. A *p* value < 0.05 was considered statistically significant in all analyses.

Descriptive statistics *n* (%) were calculated for the categorical survey data on all individual responder characteristics and applied/preferred reported management. Group comparisons were performed using chi-squared tests for nominal data and Kruskall–Wallis/Mann–Whitney *U* tests for ordinal data.

Odds ratios (ORs) including 95% confidence intervals (CIs) were calculated for dichotomous categorical variables using binary logistic regression analysis with specialism (comparing trauma surgeons with orthopaedic surgeons) as independent variable and the response (e.g. yes vs. no or surgical vs. non-surgical) as dependent variable.

Consistent with the definition employed by Sumsion regarding the Delphi survey technique, consensus was defined as ≥ 70% of agreement on a topic [17].

Due to the descriptive nature of this study, no formal sample size calculation was performed prior to data collection. It was aimed to include as many ATR-treating surgeons as possible to accurately describe the current state of practice.

## Results

### Survey responders

A total of 91 medical specialists completed the survey: 70 orthopaedic surgeons and 21 trauma surgeons. The response rate amounts to a total of 47% (70/150) of orthopaedic surgeons and 42% (21/50) of trauma surgeons based on the estimated number of eligible participants. Table 1 shows the responders’ characteristics. No statistically significant differences were found in terms of experience or practice setting between orthopaedic and trauma surgeon responders. Individually, trauma surgeons treated significantly more ATRs per year (*p* < 0.001) than orthopaedic surgeons.

**Table 1:** Characteristics of survey responders

Characteristic	*n* (%)
Experience (years)	
0–5	18 (20)
5–10	21 (23)
10–15	15 (17)
15–20	15 (17)
> 20	22 (24)
Practice setting	
Non-academic hospital without residents	27 (30)
Non-academic hospital with residents	43 (47)
Academic hospital	16 (18)
Private clinic	4 (4)
Combination of academic and private practice	1 (1)
Number of ATRs treated/year	
< 5	29 (32)
5–15	46 (51)
15–25	11 (12)
> 25	5 (6)

### Diagnosis

Responders used physical examination, palpation of tendon gap (92%) or the Thompson test (91%) as primary diagnostic tool. Concerning imaging, US was used by 45% of responders, one responder used X-rays, one MRI, and no responders used CT to diagnose an ATR.

Subgroup analyses based on specialism, experience, practice setting or number of ATRs treated showed no significant differences in the diagnostic modalities chosen.

### Primary treatment

#### General preferred primary treatment

Surgery was the reported preferred treatment among all responders (52%). Non-surgical treatment was preferred by 34%, and 14% had no preference. One responder treated all ATRs surgically, none treated only non-surgically. Although not statistically significant, trauma surgeons tended to prefer surgical treatment more often than orthopaedic surgeons (71 vs. 45%, *p* = 0.12).

Subgroup analyses based on experience, practice setting or number of ATRs treated showed no significant differences in preferred treatment.

#### Preferred primary treatment per patient factor

Table 2 shows the preferred primary treatment based on specific clinical factors. There was consensus among responders that non-surgical treatment was preferred for patients presenting with an ASA status > 3 and patients with a sedentary lifestyle. Surgical treatment was preferred for patients who were younger (age < 40) and athletic, and when a gap size > 1 cm was present. There was no consensus on a preferred primary treatment based on BMI or chronicity of rupture. Only seven responders (8%) considered other patient-related factors such as smoking, diabetes mellitus, impaired arterial circulation of the lower extremity and rupture location (distal or midportion) for their treatment choice.

**Table 2:** Treatment preference by clinical factors

Factor	Treatment	*n* (%)
Age < 40 years	Surgical	**67 (74**)
	Non-surgical	24 (26)
Age > 40 years	Surgical	41 (45)
	Non-surgical	50 (55)
ASA < 3	Surgical	56 (62)
	Non-surgical	35 (38)
ASA > 3	Surgical	5 (5)
	Non-surgical	**86 (95)**
Athletic	Surgical	**72 (79)**
	Non-surgical	19 (21)
Sedentary	Surgical	6 (6)
	Non-surgical	**85 (94)**
BMI < 30 kg/m^2^	Surgical	58 (64)
	Non-surgical	33 (36)
BMI > 30 kg/m^2^	Surgical	33 (36)
	Non-surgical	58 (64)
Gap size < 1 cm	Surgical	46 (51)
	Non-surgical	45 (49)
Gap size > 1 cm	Surgical	**68 (75)**
	Non-surgical	23 (25)
Injury < 6 weeks old	Surgical	58 (64)
	Non-surgical	33 (36)
Injury > 6 weeks old	Surgical	38 (42)
	Non-surgical	53 (58)

Bold indicates consensus was reached among responders, (> 70%) agreed on an answer

Orthopaedic and trauma surgeons differed significantly in their preferred primary treatment of patients older than 40 (*p* = 0.02) and with ASA status < 3 (*p* = 0.04); trauma surgeons preferred surgical treatment more often for both (OR 3.19, 95% CI 1.14–8.90 and OR 3.38, 95% CI 1.03–11.07). Although not statistically significant, more trauma surgeons seemed to prefer surgical treatment for patients with a higher BMI (*p* = 0.08) and gap size < 1 cm (*p* = 0.09) than orthopaedic surgeons.

Subgroup analyses based on experience or number of ATRs treated showed no significant differences by clinical factors in preferred primary treatment.

#### Non-surgical treatment methods

The non-surgical treatment methods are presented in Table 3. There was consensus that the initial immobilisation should consist of placing the foot in a plaster cast in equinus position (90%). No consensus was found on the exact length of initial immobilisation or the time at which weight-bearing was allowed; although there was consensus for weight-bearing within 6 weeks (89%), the chosen period ranged from 2 to 12 weeks. The two most chosen durations of immobilisation were either 2 weeks (46%) or 6 weeks (38%). There was consensus that the foot position should be changed (82%) and that this should be done every 2 weeks (85%).

**Table 3:** Methods of non-surgical and surgical treatment

Treatment method	Surgical *n* (%)	Non-surgical^a^*n* (%)
Preoperative antibiotics		
Yes	37 (41)	
No	54 (59)	
Preoperative anticoagulants		
Yes	38 (42)	
No	53 (58)	
Surgical technique		
Open repair	59 (65)	
Augmented repair	5 (5)	
Percutaneous	9 (10)	
Combined mini-open	17 (19)	
Other not specified	1 (1)	
Tunneling through the calcaneus		
Yes	12 (13)	
No	**79 (87)**	
Suturing technique		
Bunnell	50 (55)	
Kessler	19 (21)	
Epitendinous	2 (2)	
Mitek-anchors	7 (8)	
Krackow	2 (2)	
Other	11 (12)	
Type of sutures		
Absorbable	61(67)	
Non-absorbable	30 (33)	
Initial immobilisation		
Equinus and plaster cast	54 (59)	**81 (90)**
Non-equinus and plaster cast	24 (26)	2 (2)
Boot/brace	9 (10)	5 (6)
Tape	4 (4)	2 (2)
Length of initial immobilisation (weeks)		
2		41 (46)
3		3 (33)
4		6 (7)
5		1 (1)
6		34 (38)
> 6		5 (6)
Change foot position during immobilisation		
Yes	61 (67)	**74 (82)**
No	30 (33)	16 (18)
Frequency of foot position change^b^		
Every week	7 (11)	7 (9)
Every 2 weeks	**52 (85)**	**63 (85)**
Every 3 weeks	2 (3)	3 (4)
Every 4 weeks	0 (0)	1 (1)
When can the patient bear weight (weeks)		
2	35 (38)	30 (33)
4	14 (15)	12 (13)
6	34 (37)	39 (43)
8	4 (4)	4 (4)
10	2 (2)	2 (2)
12	1 (1)	3 (3)

Bold indicates consensus was reached among responders, (> 70%) agreed on an answer

^a^One orthopaedic surgeon did not complete the questions as he/she never treats non-surgically

^b^Eight trauma surgeons and 22 orthopaedic surgeons (surgical) and three trauma surgeons and 13 orthopaedic surgeons (non-surgical) did not answer this question as they did not change the foot position during immobilisation

Orthopaedic surgeons and trauma surgeons differed significantly on their methods of initial immobilisation (*p* = 0.001), with trauma surgeons prescribing methods other than a plaster cast more often (24 vs. 6%). Although not statistically significant, trauma surgeons tended to prescribe a shorter length of initial immobilisation (*p* = 0.07), with 72% of trauma surgeons immobilising for ≤ 4 weeks compared to 51% of orthopaedic surgeons. Responders with more experience recommended a longer period until the patient could bear weight (*p* = 0.002), with 72% of responders with ≥ 15 years’ experience recommending weight-bearing after 6 weeks compared to 40% of those with < 15 years’ experience.

Subgroup analyses based on practice setting or number of ATRs treated showed no significant differences in non-surgical treatment methods.

### Surgical treatment methods

The surgical treatment methods are presented in Table 3. There was no consensus on the administration of preoperative antibiotics and anticoagulants, surgical technique, suturing type and methods or postsurgical immobilisation methods. Open repair (65%) was the most common surgical technique and Bunnell sutures (55%) were mostly applied. The postoperative immobilisation method was the same as that chosen for non-surgical: plaster cast with the foot in equinus position (59%).

Trauma surgeons and orthopaedic surgeons differed significantly on surgical technique (*p* = 0.001), suturing technique (*p* = 0.002) and initial immobilisation methods (*p* < 0.001). The most frequent choices of trauma surgeons were minimally invasive techniques [combined mini-open (48%) and percutaneous (14%)], with orthopaedic surgeons gravitating towards open repair techniques (73%). Trauma surgeons used bone anchors more often (24 vs. 3%), and orthopaedic surgeons Bunnell sutures (61 vs. 33%). Trauma surgeons prescribed a brace as initial postoperative immobilisation method more often than orthopaedic surgeons (29 vs. 4%).

Subgroup analyses based on practice setting or number of ATRs treated showed no significant differences in surgical treatment methods.

### Rehabilitation

Table 4 shows that responders differed greatly on rehabilitation methods after surgical and non-surgical treatment. The type of protection varied, with heel lifts and walking boots as the most commonly used. Although there was consensus among responders to refer their patients to physiotherapists, trauma surgeons were significantly less likely to refer patients to physiotherapy after surgery than orthopaedic surgeons (OR 0.23; 95% CI 0.06–0.83). Tendon healing was primarily monitored via patient follow-up, one responder used US and none used MRI to monitor healing. There were no significant differences in rehabilitation methods after surgical and non-surgical treatment.

**Table 4:** Rehabilitation methods

Rehabilitation method	Surgical *n* (%)	Non-surgical^a^*n* (%)
Applied protection		
Walking boot	23 (25)	27 (30)
Heel-lift	31 (34)	28 (31)
Brace	4 (4)	15 (17)
Tape	14 (15)	14 (16)
None	19 (21)	5 (6)
Not specified		1 (1)
Referral to physiotherapist		
Yes	**79 (87)**	**79 (88)**
No	12 (13)	11 (12)
Monitoring modality		
Patient follow-up	**85 (93)**	**84 (92)**
Questionnaires	5 (6)	4 (4)
Heel-rise test	17 (19)	17 (19)
Tolerated load on tendon	16 (18)	15 (17)
US	0 (0)	1 (1)
MRI	0 (0)	0 (0)

Bold indicates consensus was reached among responders, (> 70%) agreed on an answer

^a^One orthopaedic surgeon did not answer the questions as he/she never treats non-surgically

Subgroup analyses based on experience, practice setting or number of ATRs treated showed no significant differences in methods of rehabilitation after non-surgical treatment or surgery.

**Fig. 1:**
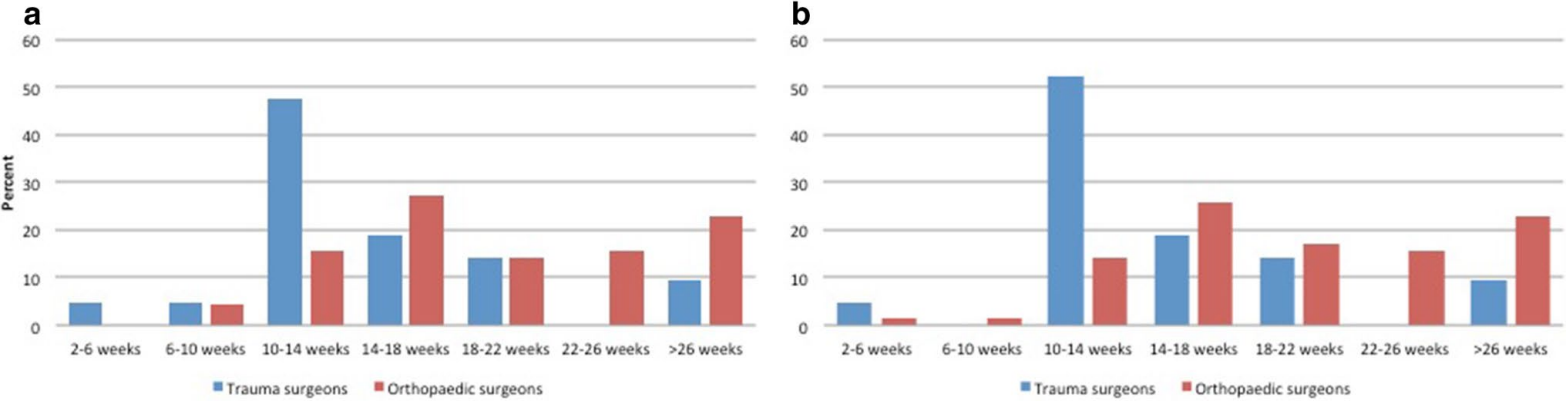
Advised time to RTS after **a** surgical, **b** non-surgical treatment

The advised time to commence RTS after both surgery and non-surgical treatment ranged from 2 to 6 weeks to > 26 weeks after initial injury, with 14–18 weeks as most responded answer. There was no significant difference in advised time to RTS after surgical or non-surgical treatment. Figure 1 shows trauma and orthopaedic surgeons differed significantly on advised time to RTS after surgical (*p* = 0.002) and non-surgical treatment (*p* = 0.001). Orthopaedic surgeons recommended a longer period until patients could RTS.

Subgroup analyses based on experience, practice setting or number of ATRs treated showed no significant differences in advised time to return to sport.

## Discussion

The most important finding of this present study was the variation in care of ATRs in the Netherlands provided by orthopaedic and trauma surgeons. To our knowledge this is the first study to gather such information and to describe the entire ATR management applied from diagnosis to rehabilitation.

This description showed considerable practice variation and a lack of consensus on ATR management, particularly concerning management in the perioperative and rehabilitation phases. There are also significant differences in applied/preferred management of ATRs between orthopaedic and trauma surgeons. Orthopaedic surgeons tended to prefer non-surgical treatment more often, followed a different perioperative protocol and advised a longer period before patients could return to sport. Lastly, management—especially concerning time to weight-bearing as well as preferred primary treatment for specific patient groups—is not in concordance with recent scientific evidence and clinical guidelines.

Although only an estimated response rate was provided, we consider the sample of this study a good representation of ATR-treating orthopaedic surgeons and trauma surgeons in the Netherlands. Due to subspecialisations in the Dutch healthcare system, usually one or two surgeons per hospital or department treat ATRs (foot/ankle and/or sport-injury specialists in their respective practices). Given the total number of eight academic hospitals and 83 general hospitals in the Netherlands [18], we estimate that the ATR-treating specialists in at least 60% of all practice settings are represented in the results. Multiplying the median number of ATRs treated by the responders (Table 1) amounts to roughly 900 ATRs per year. This is equivalent to approximately 80% of all ATRs presenting in the Netherlands. We, therefore, consider the 91 responses that we analysed to accurately reflect current ATR management in the Netherlands. This is a higher absolute response number than described by the two other studies that surveyed individual specialists (in the UK) on acute ATR management [15, 19].

### Current practice

#### Diagnostics

There is consensus on the diagnosis and the diagnostic tools used in ATRs: responders relied on the physical examination rather than imaging. This low dependence on imaging is likely due to the ease and high diagnostic accuracy of clinical tests [20] as well as the ‘typical’ ATR presentation involving sports trauma, an audible snap and/or a feeling of being kicked. In addition, recent surveys show that Dutch orthopaedic surgeons see no additional value in diagnostic musculoskeletal imaging [21]. This preference adheres to the recommendations made by the AAOS and a recent systematic review for a comprehensive physical examination of ATR patients [13, 22].

#### Primary treatment

In terms of primary treatment, responders agreed on non-surgical management for systemically diseased (ASA > 3) and sedentary patients with an ATR. The recommendation made in the AAOS guidelines to ‘cautiously approach surgery in the sedentary, obese and systemically diseased’ was adhered to [13]. In this study, obesity (BMI > 30 kg/m^2^) was not reported to be a clear indication for non-surgical treatment. Obesity is a significant risk factor for developing an ATR [7, 23] as well as for infection and other complications following orthopaedic surgery [24]. The lack of consensus on the treatment of obese patients requires additional awareness of the AAOS guideline and careful consideration by surgeons treating ATRs.

There was consensus that surgery is the preferred treatment for athletic patients, as is also preferred by Scandinavian and British specialists and supported by the literature [14, 15, 25, 26]. The agreement among the responders for surgical treatment of ATRs with a larger tendon gap size (> 1 cm) adheres to recent scientific evidence showing non-surgical managed patients with a gap size of > 1 cm to have a worse (functional) outcome [27]. Future research, however, should establish at which specific gap size non-surgical treatment should be considered, as prospective studies have employed various ultrasonographically measured gap sizes (5 mm and 1 cm) without sufficient evidence to support a specific cutoff point [25, 28, 29].

Literature has defined ATRs older than 4 weeks as ‘chronic’ [30]. These undiagnosed or untreated ruptures cause considerable morbidity and require special attention. The results showed that there is no consensus on management of these ruptures (> 6 weeks old), as responders supported both surgical and non-surgical treatments. This inconclusiveness is in line with the literature, supporting both treatments [31, 32]. To date there is insufficient evidence for clear treatment indications; future research should examine both treatment modalities for chronic ruptures.

In contrast to practice in other countries, Dutch surgeons did not generally prefer non-surgical treatment [33, 34], yet consensus was found on most of its primary methods. The protocol for non-surgical ATR treatment was similar to the SMART protocol proposed by Hutchison et al. [28], who also emphasised initial immobilisation in equinus position with casting for 2 weeks followed by gradual regression of foot position and physiotherapy. The SMART protocol resulted in the lowest rate of re-ruptures to date, as well as excellent patient-reported outcomes [28]. Despite these promising results, Dutch surgeons seem not to adhere to this protocol entirely and prescribed either 2 or 6 weeks of initial immobilisation. This prescribed period is dissimilar to the 1-week period applied in recent Dutch papers [35, 36]. We recommend further adherence to this non-surgical treatment protocol with short casting (maximum 2 weeks), followed by careful weight-bearing.

Responders generally preferred surgical treatment, yet there was a lack of consensus on perioperative methods. This might be due to a dearth of scientific evidence: a systematic review yields that only four trials have compared open and less invasive techniques, showing no significant difference in outcome and insufficient evidence comparing suturing methods [37]. We hypothesise that the preference for surgery among responders is due to the influence of several Dutch studies showing the reliable results of a minimally invasive technique [38, 39] and its superiority to non-surgical treatment in terms of complications [36]. Nonetheless, we recommend that Dutch surgeons re-evaluate their general preference for surgical treatment, as recent evidence establishing that non-surgical treatment that shows similar results has produced an international rise (in Scandinavia and North America) in the use of non-surgical ATR treatment [3, 33, 34, 40]. There is consensus on postoperative immobilisation after ATR repair, probably based on the assumption that early mobilisation puts patients at risk for re-ruptures. Several systematic reviews, however, have determined immobilisation may not be necessary or helpful at all, and that early dynamic rehabilitation using a brace results in safe results with higher patient satisfaction [41]^;^ superior, more rapid recovery [42]; and a quicker return to sporting activities [43]. A trend towards minimal immobilisation (2 weeks) is also advocated after peroneal tendon repair [44]. Similarly to non-surgical treatment we therefore recommend weight-bearing as early as possible after surgery, as already proposed by other Dutch authors [36, 45].

#### Rehabilitation

Opinions and usual care concerning the rehabilitation phase varied, and practice seemed to be based on the individual decisions. This is in line with previous research on ATR rehabilitation practice in the UK [46]. Although early weight-bearing (< 2 weeks) after surgery is associated with fewer complications and better functional recovery [27, 41, 42, 47–49], only 39% of specialists allow for weight-bearing within 2 weeks of surgery and 33% after non-surgical treatment. This study showed that a many Dutch surgeons (49%) do not adhere to the AAOS recommendations of early mobilisation (2–4 weeks). This contrasts the practice of Scandinavian surgeons, 83–100% of whom allow weight-bearing within 4 weeks [14] as well as the weight-bearing protocols proposed in Dutch publications [36, 38, 39, 45].

The protection measures used during rehabilitation varied. This was also reported in the UK [46] and is in line with the lack of evidence in the literature. With the availability of novel orthoses and increasing use of functional rehabilitation more high-quality research on rehabilitation protection is required.

There was consensus to refer patients to physiotherapists. Qualitative research has shown that trauma patients report physiotherapy as beneficial to recovery [50], implying that this referral potentially increases patient satisfaction. Nonetheless, the Royal Dutch Society for Physiotherapy (KNGF) has no recommendations or guideline statements for physiotherapists managing rehabilitation after ATR, which further impairs uniformity in ATR rehabilitation methods in the Netherlands.

A recent systematic review on RTS following ATR reported a range of 2.9–10.4 months [51] and a mere 80% return to their pre-injury levels of physical activity at ATR [51]. Likewise, in this survey no consensus on the topic was found. Because many ATR patients are physically active and ultimately sustain their rupture during sports [9], we believe a minimum period until RTS should be determined to optimise return yet avoid performance deficits and/or re-injury. We recommend this minimum period takes into consideration the vulnerable phase in which most re-ruptures occur (6–12 weeks) [52]. Ultimately, in clinical practice RTS should be tailored to each individual patient, while also considering other factors such as their fear of re-injury and motivation for RTS, among other things. This study, therefore, concludes that a multifaceted approach involving cooperation between surgeons, sports and exercise medicine physicians, physiotherapists and sport psychologists is required.

### Responder comparison

Nearly all of the differences in ATR management between responders were based on their specialism (trauma vs. orthopaedic surgery) as opposed to experience, practice setting, or number of ATRs treated per year. Non-surgical treatment and different immobilisation and surgical methods were more likely to be used by orthopaedic surgeons. Orthopaedic surgeons generally employed more traditional methods of ATR management such as immobilisation via plaster casts and open repair with Bunnell sutures, whereas trauma surgeons were more likely to use various orthoses and minimally invasive surgical techniques. Orthopaedic surgeons were also more conservative in their advised period of return to sport, advising a longer wait.

At first glance, these differences in usual practice between trauma and orthopaedic surgeons could be explained by the structure of the Dutch healthcare system, where orthopaedic surgery and trauma surgery are different specialisms following distinct medical training, yet both treating trauma patients. As a prior survey on the treatment of ankle fractures in the Netherlands showed few or no differences between orthopaedic and trauma surgeons [53], the lack of guidelines is most probably what underlies differences in ATR management between the two treating specialisms. Perhaps each specialism has separate guidelines written by individual experts with a limited following, hence the differences in management.

The clinical relevance of this study is that it allows clinicians in the Netherlands and abroad to reflect on and compare their current ATR practice, guide treatment decisions and define future (research) directions. The findings suggest that to develop clinical guidelines, multidisciplinary collaboration is required; this warrants the cooperation of orthopaedic surgeons and trauma surgeons in the Netherlands.

## Conclusion

This study provides insight into current management of ATR in the Netherlands. The diagnosis of ATR and indications for primary non-surgical or surgical treatment based on clinical factors are only partially in concordance with the limited available scientific evidence and guidelines. There is a general lack of consensus among individual specialists as well as significant differences in preferred and applied management between Dutch orthopaedic surgeons and trauma surgeons. The use of existing evidence, the development of clinical guidelines for primary treatment of different patient populations and the application of evidence-based rehabilitation principles should be encouraged.

## Electronic supplementary material

Below is the link to the electronic supplementary material.

Supplementary material 1 (DOCX 26 KB)
