# Supplementary material for: Surveying the management of Achilles tendon ruptures in the Netherlands: lack of consensus and need for treatment guidelines
Source: Knee Surg Sports Traumatol Arthrosc. 2018 Jul 3;27(9):2754–64. doi: 10.1007/s00167-018-5049-5 (PMC6706364; doi:10.1007/s00167-018-5049-5)
Supplement: Supplementary file 1 — Supplementary material 1 (DOCX 26 KB) [file 167_2018_5049_MOESM1_ESM.docx]

**Survey Questions**

**General questions:**

I am:

1. a trauma surgeon
2. an orthopaedic surgeon
3. a sports and exercise medicine physician
4. other (please specify):

How many years of practice experience do you have?

1. 0-5
2. 5-10
3. 10-15
4. 15-20
5. >20

I work in:

1. a non-academic hospital WITHOUT residents
2. a non-academic hospital WITH residents
3. an academic hospital
4. other (please specify):

Does your practice setting have specific guidelines for management of Achilles tendon ruptures?

1. Yes
2. No

How many Achilles tendon rupture treatments are you involved in per year?

1. <5
2. 5-15
3. 15-25
4. >25

Which tests do you use for the diagnosis and treatment planning of an Achilles tendon rupture? (multiple answers possible)

1. Palpation of tendon gap
2. Thompson test
3. X-ray
4. Ultrasound
5. CT
6. MRI
7. Other (please specify):

Please list any other tests you use here:

Which treatment do you prefer in the presence of the following clinical factors?

| Factor | Surgical | Non-surgical | Factor | Surgical | Non-Surgical |
| --- | --- | --- | --- | --- | --- |
| Age <40 |  |  | Age >40 |  |  |
| ASA <3 |  |  | ASA >3 |  |  |
| Athletic patient |  |  | Sedentary patient |  |  |
| BMI <30 |  |  | BMI >30 |  |  |
| Gap size <1 cm |  |  | Gap size >1 cm |  |  |
| Injury <6 weeks old |  |  | Injury >6 weeks old |  |  |

Are your patients generally satisfied with the treatment and recovery?

1. Yes
2. No

Which treatment do you generally prefer?

1. Surgery
2. Non-surgical
3. I only treat surgically
4. I only treat non-surgically
5. No preference

**Questions about surgical treatment:**

Which surgical method do you use?

1. Open repair
2. Open repair + augmentation
3. Percutaneous
4. Arthroscopic
5. Combined mini-open
6. Other (please specify):

Which suturing method you apply?

1. Bunnel
2. Kessler
3. Kleiners
4. Epitendinous
5. Mitek anchors
6. Other (please specify):

Do you perform tunnel suturing through the calcaneus?

1. Yes
2. No

What type of suturing material do you use?

1. Resorbable
2. Non-resorbable

Do you administer preoperative anticoagulants?

1. Yes
2. No

Do you administer preoperative antibiotics?

1. Yes
2. No

Which of the following best describes your methods of initial immobilisation?

1. Plaster cast with foot in equinus position
2. Plaster cast with foot in non-equinus position
3. Tape
4. Brace
5. Other (please specify):

If you use another method of immobilisation, please list it here:

Do you change the foot position, and if so what is the frequency?

1. Every week
2. Every 2 weeks
3. Every 3 weeks
4. Every 4 weeks
5. Less than every 4 weeks
6. I do not change the foot position

How long before you allow your patients to bear weight?

1. 2 weeks
2. 4 weeks
3. 6 weeks
4. 8 weeks
5. 10 weeks
6. 12 weeks
7. > 12 weeks

What protection do you prescribe six weeks postoperatively?

1. Walking boot
2. Brace
3. Tape
4. Heel lift
5. Other (please specify):

Do you refer to a physiotherapist?

1. Yes
2. No

What is your advice concerning period of return to sports?

1. 2-6 weeks
2. 6-10 weeks
3. 10-14 weeks
4. 14-18 weeks
5. 18-22 weeks
6. 22-26 weeks
7. >26 weeks

How do you monitor recovery during the rehabilitation phase? (multiple answers possible)

1. Ultrasound
2. MRI
3. Heel-rise endurance
4. Tolerated tendon load
5. Questionnaires
6. Patient follow-up
7. Physiotherapist follow-up
8. Other (please specify):

**Questions about non-surgical treatment:**

Which of the following best describes your methods of initial immobilisation?

1. Plaster cast with foot in equinus position
2. Plaster cast with foot in non-equinus position
3. Tape
4. Brace
5. Other (please specify):

How long is this immobilisation period?

1. 2 weeks
2. 3 weeks
3. 4 weeks
4. 5 weeks
5. 6 weeks
6. > 6 weeks

Do you change the foot position, and if so how often?

1. Every week
2. Every 2 weeks
3. Every 3 weeks
4. Every 4 weeks
5. Less than every 4 weeks
6. I do not change the foot position

How long before you allow your patients to bear weight?

1. 2 weeks
2. 4 weeks
3. 6 weeks
4. 8 weeks
5. 10 weeks
6. 12 weeks
7. > 12 weeks

What protection do you prescribe six weeks postoperatively?

1. Walking boot
2. Brace
3. Tape
4. Heel-lift
5. Other (please specify):

Do you refer to a physiotherapist?

1. Yes
2. No

What is your advice concerning period of return to sports?

1. 2-6 weeks
2. 6-10 weeks
3. 10-14 weeks
4. 14-18 weeks
5. 18-22 weeks
6. 22-26 weeks
7. >26 weeks

How do you monitor recovery during the rehabilitation phase? (multiple answers possible)

1. Ultrasound
2. MRI
3. Heel-rise endurance
4. Tolerated tendon load
5. Questionnaires
6. Patient follow-up
7. Physiotherapist follow-up
8. Other (please specify):
